# A Role for Strain Differences in Waveforms of Ultrasonic Vocalizations during Male–Female Interaction

**DOI:** 10.1371/journal.pone.0022093

**Published:** 2011-07-27

**Authors:** Hiroki Sugimoto, Shota Okabe, Masahiro Kato, Nobuyoshi Koshida, Toshihiko Shiroishi, Kazutaka Mogi, Takefumi Kikusui, Tsuyoshi Koide

**Affiliations:** 1 Mouse Genomics Resource Laboratory, National Institute of Genetics, Mishima, Shizuoka-ken, Japan; 2 Companion Animal Research, Azabu University, Sagamihara, Kanagawa-ken, Japan; 3 Kato Acoustics Consulting Office, Yokohama, Japan; 4 Division of Electronic and Information Engineering, Graduate School, Tokyo University of Agriculture and Technology, Koganei, Tokyo, Japan; 5 Mammalian Genetics Laboratory, National Institute of Genetics, Mishima, Shizuoka-ken, Japan; 6 Transdisciplinary Research Integration Center, Research Organization of Information and Systems, Tokyo, Japan; 7 Department of Genetics, The Graduate University for Advanced Studies (SOKENDAI), Hayama, Kanagawa, Japan; Cajal Institute, Consejo Superior de Investigaciones Científicas, Spain

## Abstract

Male mice emit ultrasonic vocalizations (USVs) towards females during male–female interaction. It has been reported that USVs of adult male mice have the capability of attracting females. Although the waveform pattern of USVs is affected by genetic background, differences among strains with respect to USV and the effects of these differences on courtship behavior have not been analyzed fully. We analyzed USV patterns, as well as actual social behavior during USV recording, in 13 inbred mouse strains, which included laboratory and wild-derived strains. Significant effects of strain were observed for the frequency of USV emission, duration, and frequency of the waveform category. Principal component (PC) analysis showed that PC1 was related to frequency and duration, and PC2–4 were related to each waveform. In the comparison of USV patterns and behaviors among strains, wild-derived KJR mice displayed the highest scores for PC2–4, and female mice paired with KJR males did not emit rejection-related click sounds. It is assumed that the waveforms emitted by KJR males have a positive effect in male–female interaction. Therefore, we extracted waveforms in PC2–4 from the USV recordings of KJR mice to produce a sound file, "HIGH2-4". As a negative control, another sound file ("LOW2-4") was created by extracting waveforms in PC2-4 from strains with low scores for these components. In the playback experiments using these sound files, female mice were attracted to the speaker that played HIGH2-4 but not the speaker that played LOW2-4. These results highlight the role of strain differences in the waveforms of male USVs during male–female interaction. The results indicated that female mice use male USVs as information when selecting a suitable mate.

## Introduction

Social communication between conspecific animals is one of the behaviors that are essential for survival and breeding. A number of reports have shown that mice use ultrasonic vocalization (USV) in several social contexts (reviewed in [1]) [2]. For example, pups that have been isolated from the mother mouse emit ultrasonic signals to attract the mother [3]. Adult mice emit ultrasonic signals during female–female and male–male interactions. It has been reported that the number of USVs emitted correlates positively with the duration of social investigation behavior [4], [5]. In addition, male mice emit a complex pattern of USVs during courtship and/or mating behavior, but female mice rarely emit USVs during male–female interaction [6]. It has been reported that female mice prefer vocalizing males to devocalized male mice during premating behavior [7]. In addition, female mice spend a longer time beside speakers that are playing male USVs than beside those playing white noise or artificial USVs [8]. Although its effect on mating success is unknown, male USV attracts females and is thought to play an important role in courtship behavior. Recently, in a playback experiment that involved wild mice, it was shown that female mice prefer the USVs of unfamiliar males to those of familiar males [9]. These results suggest that differences exist in USVs and that female mice can discriminate patterns of USVs.

It has been reported that the genetic background of a mouse affects the duration, number of calls, and frequency of their USVs [10]. In addition to characteristics such as the number and duration of calls, how frequently each categorized waveform of USV is emitted differs among laboratory strains [11], [12]. Thus, each mouse strain emits a characteristic pattern of USVs, which is determined by their genetic background. However, the evolutionary and behavioral role of these differences in USV patterns with respect to male–female interaction behavior has not been studied. It is expected that an extensive characterization of the USV repertoire in a variety of mouse strains will help to establish the role of different patterns of USVs in mouse courtship behavior. To date, USV patterns have only been characterized in mouse strains that belong to the Domesticus subspecies group; most laboratory strains have been classified genetically into this group. It is speculated that the analysis of USV in a greater variety of genetic backgrounds will provide more useful information on evolutionary aspects of the role of USV patterns. The *Mus musculus* species can be divided into at least three major subspecies groups, Domesticus, Musculus, and Castaneus, on the basis of genetic profiles [13], [14], [15], [16]. A series of wild-derived strains have been established from wild mice captured in different regions of the world (Table 1) [13], [14], [17]. Given that there is wide genetic diversity among the wild-derived strains, a variety of such strains is very useful in the investigation of phenotype diversity [18], [19], [20], [21].

**Table 1:** Mouse strains used in this study.

Strain	Subspecies group	Sex	Origin
C57BL/6(B6)	Domesticus	♂	Laboratory
BALB/c	Domesticus	♂	Laboratory
BFM/2	Domesticus	♂	France
PGN2	Domesticus	♂	Canada
CAST/Ei	Castaneus	♂	Thailand
HMI	Castaneus	♂	Taiwan
NJL	Musculus	♂	Denmark
BLG2	Musculus	♂	Bulgaria
CHD	Musculus	♂	China
KJR	Musculus	♂	South Korea
SWN	Musculus	♂	South Korea
MSM	Musculus	♂	Japan
JF1	Musculus	♂	Japan
MCH	Domesticus	♀	Laboratory

In this paper, we aimed to characterize the role of different USV patterns with respect to male–female interaction. First, we characterized the USV waveforms for 13 inbred mouse strains using sonogram data from USVs emitted when a male mouse encountered a female. At the same time, we recorded the behavior of the two mice with a video camera and analyzed the occurrence of rejection behavior in the females. By analyzing the characteristics of the USV waveform emitted by males in combination with rejection behavior in females, we were able to identify USV components that might promote male–female interaction. Finally, we examined whether some patterns of male USVs were more preferable to females than others in a playback experiment.

## Materials and Methods

### Ethics Statement

Mice were maintained in accordance with NIG guidelines, and all procedures were carried out with approval (No. 21–14) from the Committee for Animal Care and Use in NIG.

### Animals

Ultrasound emissions from male mice of 13 inbred strains (C57BL/6J, BALB/cAnN, BFM/2, BLG2, CAST/Ei, CHD, HMI, JF1-s^+^, KJR, MSM, NJL, PGN2, and SWN, Table 1) were recorded during male–female interaction behavior. Ten strains (PGN2, BFM/2, BLG2, NJL, CHD, HMI, SWN, KJR, JF1-s^+^, and MSM) have been established as inbred strains after 20 generations of brother–sister mating at the National Institute of Genetics (NIG, Mishima, Japan). CAST/Ei was obtained from The Jackson Laboratory, USA. BALB/cAnN (BALB/c) was obtained from the National Institutes of Health (NIH), USA. Given that JF1 mice are known to have an auditory disability, we used a spontaneous revertant, JF1-s^+^. As a consequence, the mice have a black coat color and normal auditory perception. Mice of the strains C57BL/6JJcl (B6) and MCH were purchased from CLEA Japan, Inc. (Tokyo, Japan). The B6 mice were bred at the NIG and used for the experiments. Females of hybrid mice MCH, produced by crossing four different inbred strains that originated in ICR outbred stock, were used for social interactions for recording the ultrasound signals. All animals were maintained at the NIG under a 12 h light/dark cycle (light from 8:00 to 20:00) in a temperature-controlled room (23±2°C).

### Apparatus

Ultrasonic signals were recorded using an ultrasound microphone (CM16/CMPA Condenser ultrasound microphone, Avisoft-Bioacoustics) and recorder (UltraSoundGate 116H, Avisoft-Bioacoustics). The microphone was positioned approximately 10 cm above the cage that contained the mice. At the same time, the mice were recorded with a digital video camera (Panasonic, Osaka).

### Recording procedure

Two-month-old, male mice were mated with female mice of their own strain for 1 month. After 1 month, the male mice were housed individually for 2 days. Female mice of MCH that were older than 10 weeks were injected with pregnant mare serum gonadotropin (PMSG) to control the sexual cycle. On the test day (after the males had been housed individually for 2 days), each male mouse was transferred to a small cage (12×20 cm) with its wood chip bedding , and then an MCH female mouse was introduced into the small cage. Immediately after the female had been introduced, recording of sound and video was started. Sounds and movies were recorded for a maximum of 15 min. Recording was stopped 3 min after the male started vocalizing, but if vocalization was not present, then recording was terminated after 15 min. During this test, intromission and ejaculation were not observed, because the session was a maximum of 15 min long. If the male mouse did not emit USV, it was returned to its cage, together with a female of same strain. One or a few weeks later (minimum 1 week), this male mouse was tested again. Recording was performed during the late part of the light phase, 14:00–18:00 pm.

### Data analysis

The sound data obtained were analyzed using SAS Lab Pro (Avisoft). Spectrograms were generated by a fast Fourier transformation (FFT-length 256, time window 100 %, overlap  = 50 %). The call duration and frequency at the start point, center point, end point, maximum point, and minimum point were calculated for each individual call. From these data, we assigned the call to a waveform category, as defined by Table S1.

Male–female interactions were scored from the video data for the number of occurrences of each of the following types of behavior: grooming, sniffing of genitals, body sniffing in both sexes; attacking (biting and kicking) and mounting by males; and avoidance behavior and clicks in females. These behaviors were observed for 3 min from the first USV.

### Devocalization

Devocalization of males was performed to verify the absence of USV in females under our test conditions. All animals were anesthetized with sodium pentobarbital. Male mice from the B6 strain, which vocalize frequently, were operated on, and the inferior laryngeal nerve was bilaterally sectioned. Another group of male mice were operated on in the same manner but the inferior laryngeal nerve was not sectioned; these were used as sham-operated control mice [22], [23]. Four days after surgery, USV during male–female interaction was recorded by the same procedure as described above.

### Playback experiment

We created the USV file HIGH2-4 by cutting and pasting recordings of USVs emitted by KJR mice, which is the highest scoring strain of PC2–PC4, on the basis of high factor loadings for PC2–PC4 (>0.4). Another USV file (LOW2-4) was created by extracting waveforms of USVs on the basis of characteristics of PC2–PC4 from the low score strains, B6, BFM/2 and BALB/c. We created the white-noise file by extracting background noise that did not contain any USVs emitted by mice.

We used 10-week-old MCH females for the playback experiment. Two days before the test, female MCH mice were injected with PMSG to control the estrous cycle. Subsequently, 24 hours before the test, the mice were transferred into the test room. The test box (35×20 cm, and 20 cm high) consisted of three compartments, a neutral zone (15×20 cm) and sound zones 1 and 2 (20×10 cm each) [9]. At the end of the sound zone, there were holes in the wire mesh, and speakers were set behind the mesh. We used two speakers that contained nanocrystalline silicon thermoacoustic emitters [24], [25]. Each female mouse was placed in the neutral zone of the test box and habituated to the test box for 15 min in this zone. The dividers were then removed to allow the female to explore freely in the test box, including the sound zones. Once the female mouse had investigated both speaker meshes and returned to the neutral zone, USV playback was started simultaneously from both speakers. The loudness of HIGH2-4 and LOW2-4 was −64.59±2.07 dB (mean dB at call start ± sd) and −64.01±2.44 dB, respectively. The playback test was conducted for 5 min. For the analysis, we measured the number of entries into the sound zones and the duration of investigation of the mesh. The USV file being tested was played repeatedly during the 5 min test period.

### Statistical analysis

All statistical analysis was performed using the R package (the R Foundation for Statistical Computing, http://www.R-project.org). ANOVA was used to determine the effect of strain on each USV parameter (latency, number of calls, and so on). MANOVA was used to determine the effect of strain on waveform categories. Pearson's chi-square test was used to test the effect of strain on the frequency of the USVs. PCA was used to analyze the structure of the USVs. For PCA, each data point was standardized at mean 0, variance 1. Variables with missing data were omitted, and variables that showed high correlation (R>0.9) were combined into one variable. We analyzed components responsible for more than 5% of the variance. For cluster analysis, a distance matrix was calculated by the Euclidean method and the dendrogram was constructed by the Ward method.

## Results

### USV in the male–female interaction test

**Figure 1:**
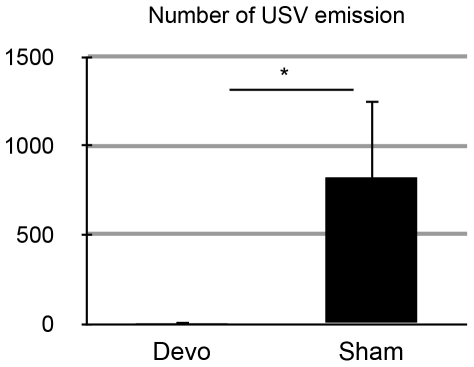
Effect of male devocalization on USV during male–female interaction. Pairs (n = 3) that comprised a female and a devocalized male displayed a significantly lower number of calls than pairs (n = 3) with sham-operated males (t-test, p<0.05). Bar indicates standard deviation. Data indicate means ± standard deviation.

In this study, we recorded USVs emitted by male mice in response to MCH females (see Materials and Methods). A single female mouse was introduced into the cage in which a male mouse was formerly placed, and the emitted USVs recorded. It has been reported that female mice do not emit USVs during interaction with males. However, we needed to confirm this finding under our experimental conditions. To examine whether the female mice emitted USVs, male B6 mice, which emit USVs frequently, were devocalized and then introduced into the same cage with an intact female mouse. We found that no USVs were detected from the females during interaction with devocalized male mice (Figure 1). In contrast, when sham-operated male mice were introduced to the female, frequent USV was detected (Figure 1). These results confirmed that male mice emitted USVs during male–female interaction but females did not emit USVs under our experimental conditions. Therefore, it can be assumed that the following USV data obtained in this study were emitted by males.

### Characterization of USV patterns in 13 inbred mouse strains

**Figure 2:**
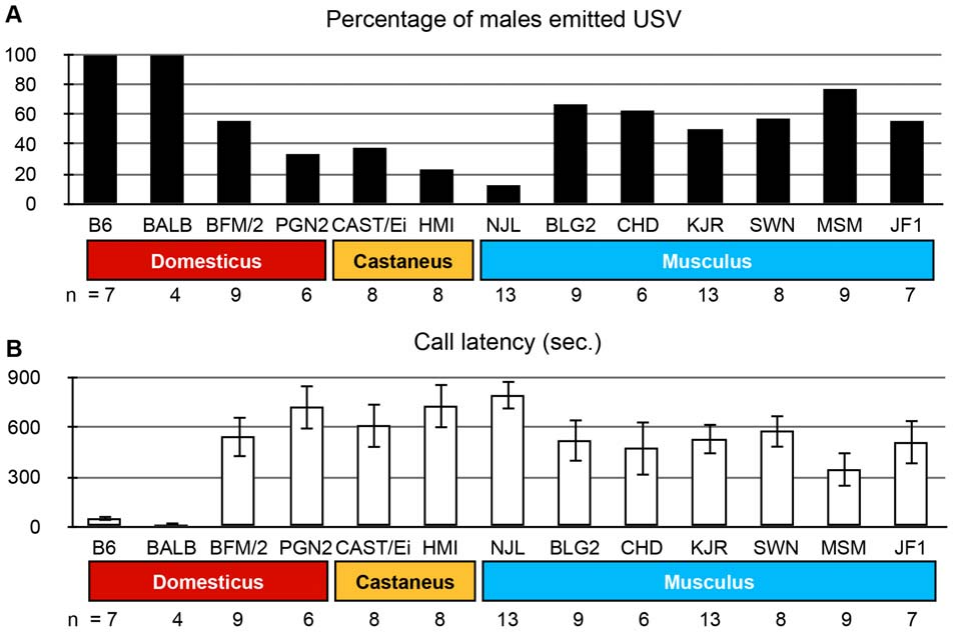
Emission of USVs. A. Percentage of males in 13 inbred mice strains that emitted USVs when they encountered a female. These percentages were not independent of strain on a chi-square test (p<0.05). B. Call latency (s.) for males from 13 inbred mice strains upon encountering a female. For individuals that did not emit any USVs, latency was recorded as 900 sec. Latency showed an effect of strain using ANOVA (p<0.001). Data indicate means ± standard error.

To study USVs emitted by males during male–female interaction, we used males from 13 inbred strains: 10 wild-derived strains, one strain derived from fancy mice, and two laboratory strains. For the female counterparts, we used MCH mice, which were produced as hybrids of four different inbred strains that originated in the ICR outbred colony. As a consequence, the MCH females displayed genetic heterogeneity among individuals, and the USVs emitted from male mice were assumed not to be specific to a certain female strain. We found that most of the wild-derived mice did not emit USVs. In particular, for PGN2, CAST/Ei, HMI, and NJL mice, USVs were emitted in fewer than 40 % of the pairs of mice we examined (Figure 2A). Even if these strains emitted USVs, the number of calls was very small. In contrast, the laboratory mouse strains (B6 and BALB/c) emitted USVs in all trials. The frequency of emission in the trials showed a strain effect (chi-square test, p<0.05). In addition, call latency, i.e. the time from an encounter with a female to emission of the first call, showed a significant effect of strain (p<0.001) on one-way analysis of variance (ANOVA), and was generally longer in wild-derived mouse strains than in laboratory mouse strains (Figure 2B).

**Figure 3:**
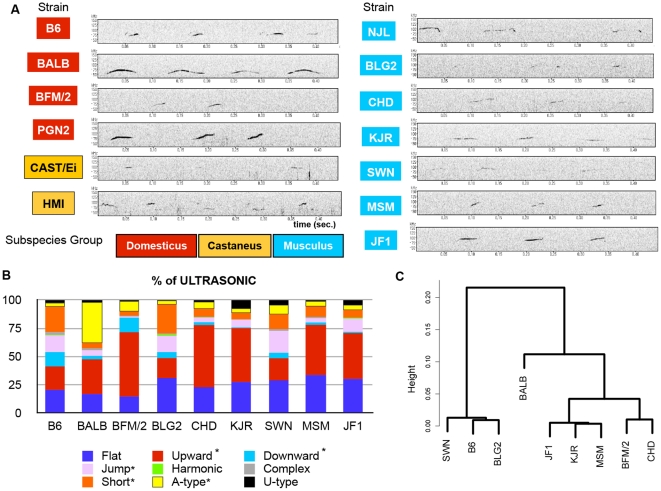
Analysis of each category of waveform. A. Representative pattern of USV. The figure shows examples of USV calls for each strain. In each panel, the *x* axis indicates duration (sec.) and the *y* axis shows frequency (kHz). B. Mean percentage compositions for each waveform category in the 13 strains. The mean of each strain was calculated using data from 3 mice. ANOVAs were performed with these data. Asterisks indicate a significant effect of strain for the waveform category in ANOVA (p<0.01). C. Cluster analysis for the mean percentage compositions for each waveform category among the different strains. The distance matrix was calculated by the Euclidean method and the dendrogram was drawn by the Ward method. This dendrogram did not reflect genetic relationships [15].

A number of mouse strains showed a characteristic pattern of USVs (Figure 3A). To characterize the pattern of USVs in more detail, we used sound data from mice who emitted more than 10 calls. Given that NJL, HMI, PGN2, and CAST/Ei mice did not emit a sufficient number of USVs for subsequent analyses, these strains were not characterized further. For the remaining nine strains, we calculated the following parameters: frequency (start point, mid point, end point, maximum point, and minimum point) and call duration. The results are summarized in Table 2. The frequency and duration of the USVs showed a significant effect of strain on ANOVA, but call latency and number of calls were not significantly different among strains. Among the nine inbred mouse strains, BALB/c mice displayed the lowest frequency and longest duration for the USVs, whereas BLG2 mice showed the shortest duration and highest frequency.

**Table 2:** Differences in ultrasonic vocalization among male mice of nine inbred strains.

Mean within strain	B6	BALB/c	BFM/2	BLG2	CHD	KJR	SWN	MSM	JF1	P values
Call latency (s.) ±s.d.	42.5±63.2	9.7±2.4	96.9±99.7	69.9±28.1	50.6±65.4	24.8±19.7	28.2±6.2	45.2±45.6	94.6±73.7	No
Number of calls±s.d.	231. ±244.9	39±367.1	69.3±27.8	168.±30.4	135±75	82±101.3	100±37.5	75±27.2	234.±146.5	No
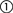 Duration (ms.) ±s.d.	17.1±4.8	37.1±8.2	23±4.3	12.7±3.9	21.8±5.1	22.6±4.5	17.7±2.7	13.9±1.4	18.5±1.8	p<0.001
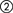 Start Frq. (kHz) ±s.d.	74±1.6	53.9±2.7	68.3±2.7	83.4±3	76.8±1.3	67±2.6	80.6±4.2	77±3.7	80.8±9.7	p<0.0001
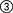 End Frq. (kHz) ±s.d.	75.4±1.5	58±4.1	75.7±3.3	85.7±2.5	83.9±2	73.8±4.4	83.9±4.5	83.3±6.1	84.9±12	p<0.001
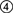 Mid Frq. (kHz) ±s.d.	74.7±0.4	60.5±3.6	75.1±3.9	85.6±2.8	81.1±1.9	70.2±3.4	83.3±3.7	81.2±5.1	83.8±10.2	p<0.0001
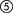 Min Frq. (kHz) ±s.d.	69.1±1.3	51.4±1.9	65.7±1.5	80.9±2.4	75.1±1.2	65.5±2.9	78.1±3.5	75.7±4	78.1±9.4	p<0.0001
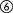 Duration until Min (ms.) ±s.d.	8.4±3.4	14.6±7	6.9±3	4.2±0.3	3.9±0.9	4±3.2	5.5±1.3	2.5±1	5.5±1.7	p<0.01
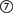 Max Frq. (kHz) ±s.d.	81.5±1.1	64.9±5.8	80.7±5.6	89.5±3	86.2±2	75.4±4.4	87.7±5	85.3±5.8	88.6±12	p<0.01
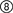 Duration until Max (ms.) ±s.d.	8.3±1.9	20±4.7	14.6±0.5	7.8±3.2	17±4.2	16.8±1.7	10.6±1.7	10.9±2.2	14.1±2.9	p<0.001

“No” means no significant effect of strain by ANOVA (p>0.05).

### Strain differences in the waveform composition of USVs

It has been reported that the USVs of mice are complexes of different sonographic components (waveforms) [11]. To characterize the structures of the USVs that were emitted by the strains investigated, we categorized the waveforms into nine types (Table S1), which is consistent with the results described in a recent report [11], with minor modification. The frequency of each waveform was calculated for each animal and analyzed in detail to investigate the characteristics of each strain. The percentage compositions of the waveforms are shown in Figure 3B. The waveform categories showed a significant effect of strain on MANOVA (p<0.0001). Moreover, the strain effect for the frequency with which each waveform category was emitted was tested by ANOVA. Upward, Downward, Jump, Short, and A-type waveforms all showed a significant effect of strain (p<0.01, 0.01, 0.05, 0.001, 0.0001, respectively). BALB/c mice showed a high percentage of A-type waveforms [12], whereas B6 and BLG2 mice showed a high percentage of Short-type calls. The main characteristics of the waveform compositions in CHD, JF1, KJR, and MSM mice appeared to be similar. To evaluate the similarity among strains, we performed cluster analysis on the basis of the waveform patterns (Figure 3C). On this basis, the strains were clustered into three groups by the Ward method. The classification of the strains into these three groups did not reflect their genetic relationships. The pattern of USVs was as different among closely related strains as among genetically remote strains. Strain differences in these waveform patterns have been reported previously [11], [12]. In addition to the strain differences with respect to waveform patterns, the duration and frequency of USVs showed significant strain effects (Table S2).

### Principal component analysis (PCA) of ultrasonic vocalization

**Figure 4:**
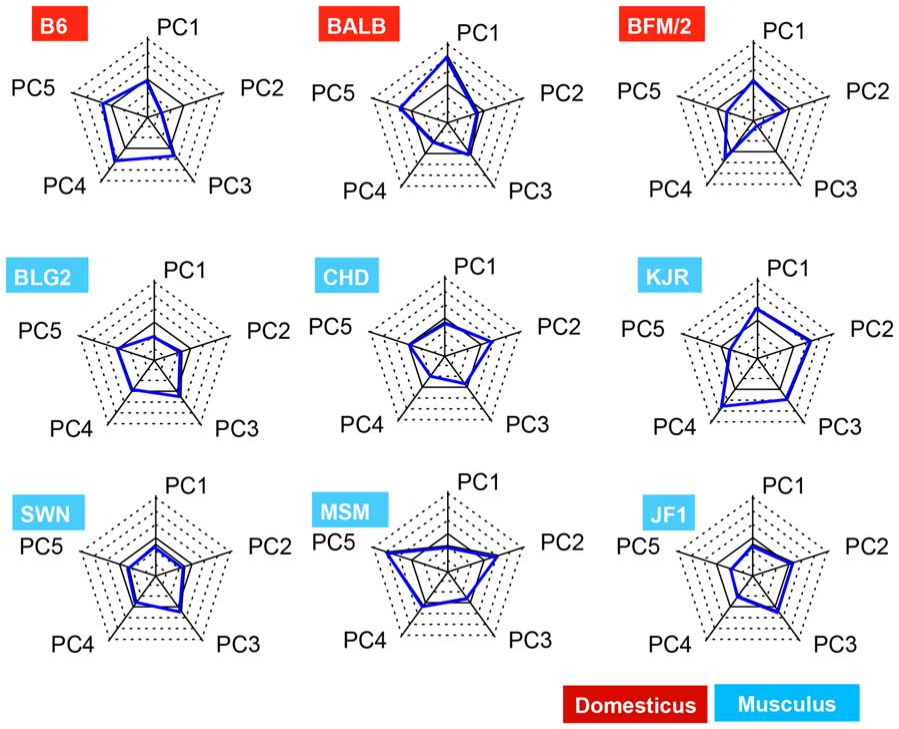
Radar charts representing the characters of the USV pattern in each strain. The value for each strain represents the standardized score for each principal component (mean  = 0, variance  = 1). The transverse solid lines in the middle of the dotted lines indicate the mean value ( = 0). Transverse dotted lines are drawn at intervals of 1× variance.

We performed PCA to simplify the differences among strains, using variables that had shown a significant strain effect in the ANOVA. In the analysis, highly correlated variables (R>0.9) were combined into one variable. More than 90% of the variance in the data was explained by principal components (PC) 1 to 5 (PC1–PC5) (Table 3). We interpreted the component that showed more than 5% proportion of variance. For PC1, the frequency at each point and duration of each waveform showed high factor loadings. Frequency and duration were negatively correlated; thus, a high score for PC1 indicated USV of high frequency with short duration. For PC2, call duration and the maximum frequency of the Flat waveform were positively correlated, but the minimum frequency was negatively correlated. For PC3, the percentage composition of Jump and the maximum frequency of U-type waveforms displayed high factor loadings. PC4 indicated duration until the maximum or minimum peak for waveforms of the Short, A-type, and Jump type. For PC5, the slope of the Downward waveform showed high factor loadings. In summary, major differences among the mouse strains occurred with respect to frequency and duration, as well as for Flat, U-type, and Downward waveforms. To clarify the differences among the nine inbred strains, we have presented the standardized scores for the five components in each strain on radar charts (Figure 4). The charts represent the character of the USV pattern in each strain. The BALB/c mice displayed a high score for PC1, which indicated calls of lower frequency and longer duration. The KJR mice displayed high scores for PC2–4 as compared with other strains. CHD and JF1 mice, and BLG2 and SWN mice, displayed similar USV patterns to each other, but the USV patterns of the BFM/2 and MSM mice were unique among the nine strains.

**Table 3:** Proportion of variance for the principal components.

Principal component		PC1	PC2	PC3	PC4	PC5	PC6	PC7	PC8
Standard Deviation		5.48	2.531	2.1705	1.8309	1.751	1.4028	0.944	0.7273
Proportion of Variance		0.59	0.126	0.0924	0.0657	0.0601	0.0386	0.0175	0.0104
Cumulative Proportion		0.59	0.715	0.8077	0.8734	0.9336	0.9722	0.9896	1
**Category**	**Factor loadings**	**PC1**	**PC2**	**PC3**	**PC4**	**PC5**	**PC6**	**PC7**	**PC8**
Flat	Duration	**0.704**	**0.573**	0.349	−0.096	−0.170	−0.100	0.077	−0.018
	Duration until Minimum peak	**0.507**	**−0.612**	0.075	−0.288	−0.116	−0.395	0.091	−0.321
	Duration until Maximum peakMaximum position	**0.502**	**0.823**	0.149	−0.139	−0.140	−0.022	0.087	−0.043
		−0.221	**0.804**	**−0.462**	−0.177	0.050	0.228	0.013	−0.071
	Minimum position	−0.079	**−0.849**	**−0.432**	0.110	−0.096	−0.198	0.034	−0.160
Short	Duration	**0.647**	−0.087	**0.504**	−0.318	−0.398	−0.000	0.223	−0.104
	Duration until Minimum peak	−0.354	−0.285	0.360	**−0.779**	−0.055	−0.210	0.099	0.016
	Duration until Maximum peak	**0.843**	0.152	0.027	0.261	**−0.436**	0.044	−0.057	−0.049
Upward	Start frequency	**−0.945**	0.061	0.217	−0.128	−0.179	−0.022	0.066	−0.047
	End frequency	**−0.974**	0.071	0.061	−0.070	−0.165	−0.021	0.090	−0.042
	Mid frequency	**−0.971**	0.049	0.059	−0.142	−0.162	0.017	0.059	−0.040
	Minimum frequency	**−0.949**	0.066	0.208	−0.113	−0.176	−0.010	0.073	−0.051
Downward	Duration	**0.889**	−0.025	−0.317	0.055	−0.317	0.046	0.017	−0.056
	Duration until Minimum peak	**0.885**	0.041	−0.378	0.078	−0.237	0.068	−0.006	−0.075
	Duration until Maximum peak	**0.902**	−0.080	−0.334	0.008	−0.241	0.100	0.005	−0.030
	Down-slope	**0.695**	−0.036	−0.025	−0.044	**−0.659**	−0.066	−0.155	0.224
A-type	Duration	**0.971**	0.112	0.054	0.110	−0.112	−0.082	0.015	0.098
	Start frequency	**−0.973**	0.001	−0.061	−0.113	−0.181	−0.053	0.042	0.020
	End frequency	**−0.962**	0.038	−0.108	−0.052	−0.195	−0.110	0.085	−0.033
	Mid frequency	**−0.915**	−0.057	−0.142	−0.037	−0.339	−0.054	0.129	0.055
	Minimum frequency	**−0.971**	0.022	−0.068	−0.073	−0.202	−0.050	0.061	−0.002
	Duration until Minimum peak	**0.729**	−0.328	0.147	**−0.492**	0.229	−0.170	−0.054	0.117
	Maximum frequency	**−0.926**	−0.085	−0.118	−0.005	−0.319	−0.093	0.100	0.021
	Duration until Maximum peak	**0.830**	0.169	0.095	0.392	−0.213	−0.220	0.131	0.091
	Up-slope	**−0.646**	**−0.493**	−0.200	0.031	0.308	**0.444**	0.055	−0.063
	Maximum position	**−0.863**	−0.087	−0.006	0.252	0.015	−0.234	0.358	−0.030
U-type	Duration	**0.920**	0.065	0.302	−0.184	0.011	0.152	0.024	0.002
	Duration until Minimum peak	**0.801**	0.282	**0.415**	0.118	0.209	0.108	0.194	−0.007
	Up-slope	−0.295	0.032	−0.053	0.255	0.363	**−0.744**	−0.393	−0.058
	Maximum position	−0.066	−0.310	**−0.808**	−0.395	−0.041	0.170	0.200	0.142
	Minimum position	−0.234	0.379	0.240	**0.414**	**0.643**	−0.165	0.361	−0.026
Jump	Duration	**0.498**	−0.339	**−0.776**	−0.085	−0.050	−0.070	0.131	0.063
	Duration until Maximum peak	**0.432**	0.132	−0.021	**−0.713**	**0.443**	−0.271	0.008	−0.130
	Start frequency	**−0.922**	0.182	−0.247	−0.060	0.096	0.170	−0.063	−0.102
	End frequency	**−0.793**	0.382	−0.218	−0.150	0.078	−0.309	−0.175	0.149
	Mid frequency	**−0.801**	0.351	−0.359	−0.202	0.167	0.176	−0.078	0.007
	Minimum frequency	**−0.817**	0.286	−0.163	0.020	0.092	0.374	−0.149	−0.230
	Maximum frequency	**−0.800**	0.096	−0.228	−0.369	0.309	−0.123	0.035	0.224
Total	Duration	**0.946**	0.026	−0.123	−0.292	−0.029	−0.041	−0.011	0.030
	Start frequency	**−0.963**	−0.019	0.180	−0.071	−0.183	−0.034	−0.026	−0.004
	End frequency	**−0.955**	0.174	0.047	−0.051	−0.211	−0.072	−0.048	0.024
	Mid frequency	**−0.966**	0.047	0.031	−0.154	−0.195	0.002	−0.030	0.016
	Minimum frequency	**−0.961**	0.082	0.166	−0.093	−0.178	−0.004	−0.040	0.029
	Duration until Minimum peak	**0.817**	**−0.489**	−0.053	−0.257	0.115	0.033	0.069	−0.074
	Maximum frequency	**−0.968**	0.008	0.020	−0.100	−0.216	−0.070	−0.029	−0.013
	Duration until Maximum peak	**0.771**	**0.484**	−0.191	−0.293	−0.167	−0.139	0.009	0.046
% category	U	0.146	**0.692**	**−0.589**	0.079	−0.130	−0.333	0.121	−0.067
	D	−0.052	**−0.757**	**−0.482**	**0.403**	−0.026	−0.169	0.002	0.001
	JM	−0.381	**−0.532**	**0.642**	−0.045	−0.253	0.148	−0.263	−0.054
	S	**−0.515**	**−0.618**	**0.429**	0.187	0.186	0.063	0.160	0.264
	A	**0.807**	−0.167	−0.092	**−0.458**	0.232	0.209	−0.057	0.044

### Analysis of behavior during male–female interactions

**Figure 5:**
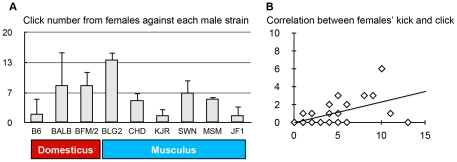
A. Number of female clicks during male–female interaction. Female clicks showed a strain effect employing ANOVA (p<0.01). Data indicate mean ± standard error (n = 3). B. Correlation between females that kicked and female clicks (R = 0.687, p<0.001, n = 102).

In the second stage of this study, we investigated the role of the differences in USV patterns with respect to male–female social interaction. ANOVA revealed no significant effect of strain on any of the behavioral components including the positive behavior of females toward males (genital sniffing and grooming; Figure S1). Thus, these social behavioral components were not significantly different among strains. ANOVA only revealed a significant strain effect (p<0.01) for clicks made by female mice (Figure 5A). A click made by a female is an audible call and is thought to be an expression of a negative reaction of the female to a male mouse [26], [27]. Indeed, we analyzed the correlation between clicks from females and kicking of males by females in all the video data, and found a positive correlation (R = 0.6868, p<0.001) (Figure 5B). This finding supports the idea that the click indicates a negative reaction of the female to a male. Thus, we used click, a negative reaction, as an opposite index of female preference for males. The KJR strain, which displayed high scores in PC2–4, triggered the fewest female clicks. Also, the PC scores PC2, PC3, and PC4 showed a weak trend for correlation with the number of female clicks (R = −0.316, −0.272, −0.278, respectively). Therefore, the PC2–4-related waveforms emitted by KJR males may have a positive effect on male–female interactions.

### Response of female mice to the playback of USVs of selected waveforms

**Figure 6:**
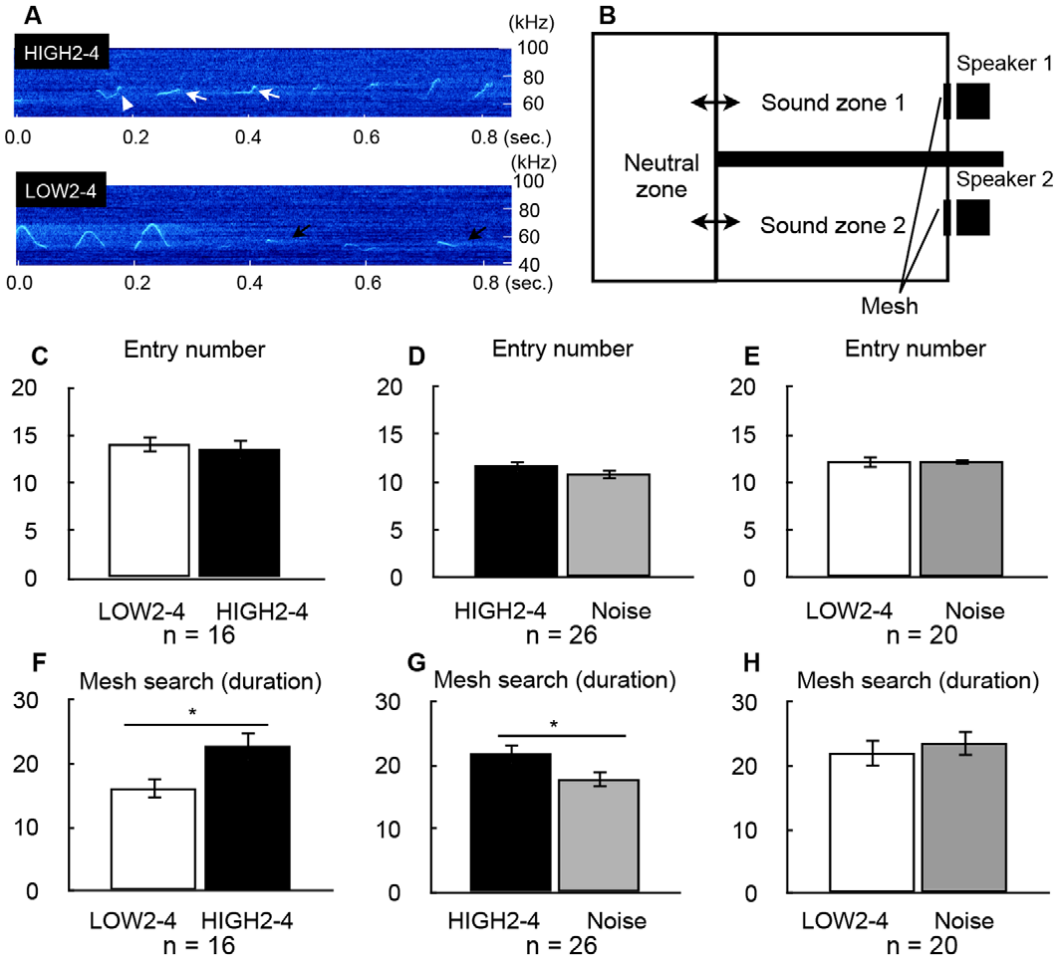
Preference of female mice for male USVs as investigated by the two-speaker method. A. Representative spectrogram of the sound files used in the playback experiments. Upper panel shows HIGH2-4, lower panel shows LOW2-4. Arrowhead indicates the reversed-J letter shape of the U-type waveform. White arrows indicate right-up flat waveform, and black arrows indicate right-down flat waveform. B. Apparatus for the playback experiment. The apparatus consisted of three chambers (a neutral zone and sound zones 1 and 2). The mouse could access the adjacent chambers through the small gates between the neutral zone and sound zones 1 and 2. Two speakers were set at the back walls and were covered with wire mesh. B and C. The two speakers played the USV files for LOW2-4 and HIGH2-4, respectively (n = 16). D and E. The speakers played the USV files for HIGH2-4 and white noise, respectively (n = 20). F and G. The speakers played the USV files for LOW2-4 and white noise, respectively (n = 26). Data are means ± standard error. Asterisks indicate significant differences between two sounds (paired t-test, p<0.05).

To examine whether the pattern of USVs could be correlated with any sign of preference or aversion in females, playback experiments using two USV files that had been created by cutting and pasting were conducted. We extracted waveforms on the basis of the factor loadings for PC2–4 from USVs emitted by KJR mice and created a sound file that was named "HIGH2-4". As a negative control, we produced another sound file, "LOW2-4", which was created by extracting waveforms in PC2–4 from the strains with the lowest scores for these components (see Materials and Methods). As a consequence, HIGH2-4 included Flat waveforms with gradually increasing frequency, U-type waveforms that were similar to the shape of a reversed letter ”J„, and Jump waveforms that jumped downwards (Figure 6A). The LOW2-4 file included Flat waveforms with gradually decreasing frequency, U-type waveforms that were similar to the shape of the letter ”J„, and Jump waveforms that jumped upwards (Figure 6A). In the playback experiment, we used two speakers ([9], Figure 6B) to play the different files to evaluate whether the female mice preferred HIGH2-4 and avoided LOW2-4.

In the first experiment, HIGH2-4 and LOW2-4 were played at the same time, one in each of the two speaker zones, to allow the mouse to choose one of the USV patterns (Figure 6A). We measured two parameters: number of entries into the sound zones (Figure 6C,D,E) and duration of contact with the mesh of the speaker (Figure 6F,G,H). The number of entries did not show a significant difference between HIGH2-4 and LOW2-4 (Figure 6C). However, the duration of contact with the mesh of the speaker did display a significant difference, and females clearly preferred HIGH2-4 (Figure 6F).

In the second experiment, we tested the preference of female mice for white noise against HIGH2-4, or white noise against LOW2-4 (Figure 6D,E,G,H). In the case of white noise and HIGH2-4, female mice showed a significantly longer duration of contact with the HIGH2-4 speaker than with the white noise speaker (Figure 6G). In contrast, a significant difference was not observed between LOW2-4 and white noise (Figure 6H). These results showed that female mice prefer HIGH2-4 to white noise, but not LOW2-4. The LOW2-4 USV had a similar effect to white noise and therefore might not have a strong aversive effect on females. These results highlight the importance of differences in the waveforms of USVs with respect to the preference of females for males of certain strains.

## Discussion

### Strain differences in USV patterns

We have shown that the degree of difference with respect to USV patterns among strains of mice did not correlate with genetic distance, as established using polymorphisms of nuclear genes [15]. Indeed, we observed that pairs of Korean strains, KJR and SWN, and Japanese strains, JF1 and MSM, showed clear differences within the pairs with respect to USV patterns. These results suggest that differences in USVs are caused by genetic drift in each local area, as was proposed in the case of singing mice [28].

There were major differences between laboratory mice and wild-derived mice with respect to call latency and percentage of mice who emitted USVs. The results indicated that laboratory strains emitted USVs much more readily than wild-derived strains. In song birds, clear differences in singing pattern have been reported between wild and domestic birds [29]. The song of Bengalese finch (*Lonchura striata* var. *domestica*), a domesticated strain, is much more complex than that of the wild strain, the white-rumped munia (*Lonchura striata*). This complexity of male songs in domesticated birds is thought to result from artificial selection for reproductive advantage [29]. In general, domestication is accompanied by intentional or unintentional selection for enhanced reproduction [30]. In laboratory mice (ICR/Alb), the percentage of mating pairs that gave birth to litters was higher in domesticated mice than in wild stock [31]. Thus, it is possible that the greater occurrence of USV in the laboratory strains B6 and BALB/c might result from unintentional positive selection for enhanced reproduction. If so, the USVs of males of domesticated strains might have a greater positive effect on the reproductive behavior of females than those of wild-derived strains. Further studies are necessary to investigate the effect of less frequent USV on reproductive behavior in wild-derived strains.

### A role for strain differences in mating behavior

In this study, we characterized differences among 13 inbred strains with respect to the waveform patterns of male USVs, as well as the social behavior of males and females during the recording of the USVs. By analyzing video images as well as sound data, we found that clicks emitted by females correlated with avoidance behavior in the females in response to the males. We speculate that this avoidance behavior in females results from a composite effect of USV and other behavior during male–female interaction. As a consequence, the causes of avoidance behavior exhibited in females should be considered carefully. In spite of these considerations, it is important to examine the contribution that differences in USV patterns among strains make to male–female interaction in order to understand some of the cues involved in the interaction.

In this study, we demonstrated that particular patterns of USV waveforms have a role in attracting females, although other types of waveform do not. As the correlation between click and PC1 (duration and frequency) was weak, the duration and frequency may not influence the preference of females to a large extent. To clarify this point further, we will need to create artificial USV files which are different only in waveform and not in duration and frequency. Such a playback experiment using these different artificial USV files may generate more direct results for female preference based on waveforms. Thus, the present results indicate an important role of strain differences in USV patterns with respect to the rate of success of males when searching for a mate. A recent report showed that different patterns of USV waveforms induce unique patterns of response in the neurons of the inferior colliculus, even if the waveforms of USVs are very similar to each other [32]. Thus, subtle differences in waveform patterns might be able to affect the choice of mate through differences in activation of the inferior colliculus. It might be interesting to investigate the activities of these neurons in response to the USV files HIGH2-4 and LOW 2-4 in the future.

It has been reported that mice tend to avoid inbreeding through discrimination on the basis of USVs [9]. Playback experiments have shown that female mice prefer an unfamiliar male USV pattern [9]. In the present study, the number of clicks made by the MCH females (Domesticus group) differed clearly between male B6 and BALB/c mice (both of which are laboratory strains that belong to the Domesticus group), between KJR and SWN mice (both of Korean origin), and between JF1 and MSM mice (both of Japanese origin). Therefore, the degree of female preference for a USV pattern is not a direct measure of the genetic distance between the male and female, but is more important in avoiding the selection of close relatives as the mating partner.

In this study, we investigated USVs during male–female social interaction behavior. Our results did not indicate any role for USVs in the actual mating behavior of mice. It has been reported that bird song changes the physiological state and mating behavior of female birds, and thus contributes to mating success [33]. It is possible that USVs emitted by male mice during male–female interaction influence the subsequent mating behaviors. Further in depth studies on the role of differences in USVs in mating behavior will lead to better understanding of the role of vocal communication in the mating behavior of mice.

## Supporting Information

Figure S1 **Behavior during male–female interactions.** A. Behavior of males. B. Behavior of females. Data indicate means ± standard error.
(PDF)

Table S1 Criteria of classification for ultrasonic vocalization patterns.
(PDF)

Table S2 P-values from ANOVA for variables of each waveform category.
(PDF)
