# Supplementary figures and images for: A Role for Strain Differences in Waveforms of Ultrasonic Vocalizations during Male–Female Interaction
Source: PLoS One. 2011 Jul 27;6(7):e22093. doi: 10.1371/journal.pone.0022093 (PMC3144874; doi:10.1371/journal.pone.0022093)

**A****Male behavior**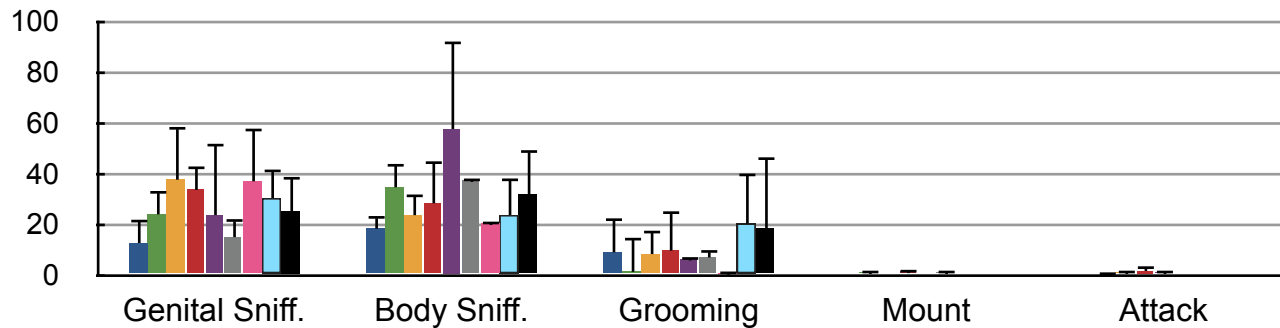**B****Female behavior**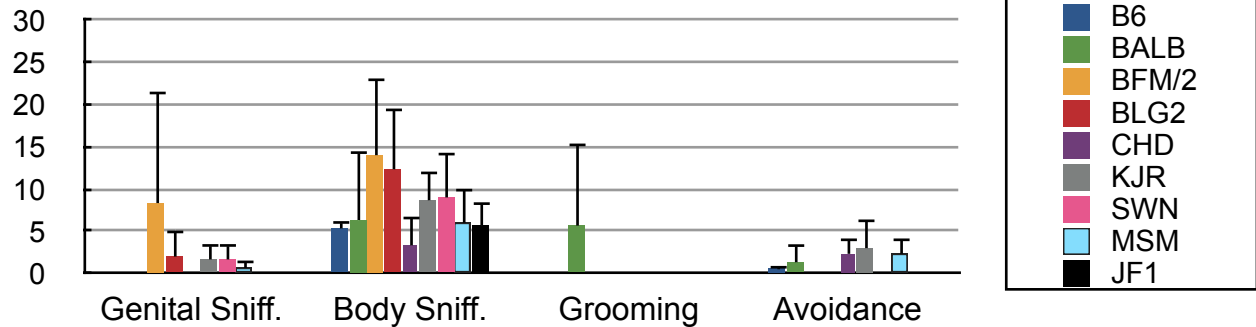

Figure S1

Supplement: Figure S1 — Behavior during male–female interactions. A. Behavior of males. B. Behavior of females. Data indicate means ± standard error. (PDF) [file pone.0022093.s001.pdf]
