# Supplementary material for: A Role for Strain Differences in Waveforms of Ultrasonic Vocalizations during Male–Female Interaction
Source: PLoS One. 2011 Jul 27;6(7):e22093. doi: 10.1371/journal.pone.0022093 (PMC3144874; doi:10.1371/journal.pone.0022093)
Supplement: Table S1 — Criteria of classification for ultrasonic vocalization patterns. (PDF) [file pone.0022093.s002.pdf]

Table S1. Criteria of classification for ultrasonic vocalization patterns.

<table>
<tr><th>Category name</th><th>Criteria</th></tr>
<tr><td>Short</td><td>< 5 ms</td></tr>
<tr><td>Flat</td><td>Max Peak–Min Peak < 6.25 kHz</td></tr>
<tr><td>Upward</td><td>End–Start ≥ 6.25 kHz, End–Start ≥ Max Peak–End</td></tr>
<tr><td>Downward</td><td>Start–End ≥ 6.25 kHz, Start–End ≥ Min–End</td></tr>
<tr><td>A-type</td><td>|Start–End| < 6.25 kHz, Max–Start ≥ 6.25 kHz, End–Start < Max–End</td></tr>
<tr><td>U-type</td><td>|Start–End | < 6.25 kHz, Start–Min ≥ 6.25 kHz, Start–End < Min–End</td></tr>
<tr><td>Complex</td><td>Two or more directional changes, each ≥ 6.25 kHz</td></tr>
<tr><td>Jump</td><td>More than one jump</td></tr>
<tr><td>Harmonic</td><td>More than two parallel horizontal lines in the spectrogram</td></tr>
</table>

| | indicate absolute value.
