# Supplementary material for: A Role for Strain Differences in Waveforms of Ultrasonic Vocalizations during Male–Female Interaction
Source: PLoS One. 2011 Jul 27;6(7):e22093. doi: 10.1371/journal.pone.0022093 (PMC3144874; doi:10.1371/journal.pone.0022093)
Supplement: Table S2 — P-values from ANOVA for variables of each waveform category. (PDF) [file pone.0022093.s003.pdf]

Table S2. P-values from ANOVA for variables of each waveform category.

Variables	Flat	Short	Upward	Downward	Harmonic	Complex	A-type	U-type	Jump
Duration	**p<0.01**	**p<0.05**	No	No	**p<0.01**	**p<0.01**	**p<0.01**	**p<0.05**	No
Start frequency	**p<0.05**	**p<0.05**	**p<0.01**	**p<0.01**	**p<0.0001**	**p<0.0001**	**p<0.01**	**p<0.001**	**p<0.001**
End frequency	**p<0.001**	**p<0.05**	**p<0.01**	**p<0.001**	**p<0.001**	**p<0.001**	**p<0.001**	**p<0.05**	**p<0.01**
Mid frequency	**p<0.01**	**p<0.05**	**p<0.01**	**p<0.01**	**p<0.0001**	**p<0.01**	**p<0.01**	**p<0.01**	**p<0.001**
Minimum frequency	**p<0.001**	**p<0.05**	**p<0.001**	**p<0.001**	**p<0.0001**	**p<0.0001**	**p<0.01**	**p<0.01**	**p<0.001**
Duration until Minimum peak	**p<0.0001**	No	No	**p<0.05**	No	No	No	**p<0.05**	No
Maximum frequency	**p<0.01**	**p<0.05**	No	**p<0.01**	**p<0.001**	**p<0.05**	**p<0.01**	No	**p<0.01**
Duration until Maximum peak	**p<0.01**	No	No	**p<0.01**	**p<0.05**	No	**p<0.01**	No	**p<0.05**
Up-slope	No	No	No	No	**p<0.01**	No	No	No	No
Max position	**p<0.01**	No	No	No	No	No	**p<0.05**	No	No
Down-slope	No	No	No	**p<0.05**	No	No	No	No	No
Min position	**p<0.01**	No	No	No	No	No	No	**p<0.05**	No

Bold indicates significant effect by ANOVA (p<0.05). “No” means nonsignificant effect of strain by ANOVA (p>0.05).
